# Temporal Filler Injection for Lifting Purposes: A Systematic Review and Meta‐Analysis

**DOI:** 10.1111/jocd.71072

**Published:** 2026-07-14

**Authors:** Amir Hashemloo, Maryam Milanifard

**Affiliations:** ^1^ Department of Medicine Shahid Beheshti University of Medical Sciences Tehran Iran; ^2^ Trauma and Injury Research Center, Student Research Committee Iran University of Medical Sciences Tehran Iran

**Keywords:** calcium hydroxyapatite, effectiveness, hyaluronic acid filler, superficial temporal artery, temporal volumization

## Abstract

**Background and Aim:**

Volume loss in the temples, which is caused by facial aging, is not aesthetically pleasing. Non‐invasive or less invasive methods have become more popular among patients and physicians. Therefore, the present systematic review and meta‐analysis aimed to determine the effectiveness of injecting various temporal fillers for lifting purposes.

**Methods:**

A search for relevant articles was conducted without time limits until June 2025 in the international databases PubMed, EMBASE, and Web of Science. The research question was developed using the Population, Intervention, Comparison, Outcome (PICO) approach to evaluate whether different dermal fillers are effective and safe in patients requiring temporal fossa augmentation. Data analyses were conducted using the meta‐analysis packages in STATA/MP version 17.

**Results:**

Subgroup meta‐analysis showed consistent effectiveness across study designs and filler types. Hyaluronic acid and calcium hydroxyapatite fillers demonstrated high effectiveness in improving temporal volume loss, with no significant differences observed between filler categories. The overall findings indicated high rates of aesthetic improvement based on the Global Aesthetic Improvement Scale (GAIS). Meta‐regression analysis suggested a significant association between injection volume and treatment effectiveness. The most commonly reported complications were mild and temporary, including edema, bruising, tenderness, erythema, and swelling at the injection site.

**Conclusion:**

Hyaluronic acid and calcium hydroxyapatite dermal fillers are effective and safe for improving temporal volume loss, with a significant association between injection volume and treatment efficacy. Further large‐scale studies are needed to refine treatment protocols and optimize outcomes.

## 1 Introduction

Age‐related atrophy of the temporalis muscle and fat layers, as well as temporal bone remodeling, leads to clinically significant volume loss in the temples [1]. The complex structure of the temporal region and the importance of its impact on facial attractiveness doubles the need for treatment with non‐invasive or minimally invasive methods. Among the minimally invasive methods that have received much attention are dermal fillers, which can increase the volume and firmness of the temporal region [2, 3]. The studies have shown that filler injections are highly effective for improving lines around the eyes, midface, and lower third of the face [4, 5, 6, 7]. Dermal fillers come in various types, such as hyaluronic acid (HA), poly‐L‐lactic acid (PLLA), and calcium hydroxyapatite (CaHA), which are used depending on the injection site and duration [8, 9, 10].

Findings related to the effectiveness of dermal fillers on the healing process of the temporal region are very sparse and under‐reported in studies, and in facial aesthetics, the temporal region is usually ignored despite its high importance in the balance and coordination of the upper part of the face [11]. To rejuvenate the face, the temporal fossa and existing lines must be treated. On the other hand, successful injection of dermal fillers in the temporal area can increase the lifting effect and give the person a more natural and youthful appearance [12, 13].

In a systematic review study, nonsurgical volumization with soft‐tissue fillers was investigated; their findings showed that temporal volume can be safely and effectively increased by injecting soft tissue fillers [14]. However, these results are related to a literature review and not the results of clinical trials or cohort studies; therefore, combining the results of clinical trials and cohort studies can provide very strong evidence. Meta‐analysis is a method that combines numerical data to provide a more comprehensive and accurate assessment. Therefore, the present systematic review and meta‐analysis aimed to determine the effectiveness of injecting various temporal fillers for lifting purposes.

## 2 Methods

### 2.1 Data Sources, Search Strategy, and Definitions

The aim of the present study was to evaluate the effectiveness of different dermal filler injections for temporal fossa augmentation using a systematic design and meta‐analysis in accordance with the PRISMA (Preferred Reporting Items for Systematic Reviews and Meta‐Analyses) guidelines [15]. An unrestricted search of the international databases PubMed, Scopus, EMBASE, Cochrane Central, and Web of Science was conducted up to June 2025 to find relevant articles. In addition, Google Scholar was also searched, and gray literature sources and clinical trial registries were screened to increase search sensitivity. In addition to using relevant keywords for the search, the references of the articles were also checked to ensure that no studies were missed.

A combination of free‐text keywords was used, including (“temporal fossa” OR “temple augmentation” OR “temporal hollowing” OR “temporal volume loss”) AND (“dermal fillers” OR “hyaluronic acid” OR “calcium hydroxyapatite”), to ensure comprehensive identification of relevant studies.

### 2.2 Study Selection: Inclusion and Exclusion Criteria

Inclusion criteria included patients who needed temporal fossa augmentation; dermal fillers were considered as the intervention group and a control group without treatment or a single‐arm study; safety and efficacy were also considered when analyzing the data and presenting overall efficacy. This section was created using the PICO approach. Current study question: Does the use of various dermal fillers (intervention (I)) in patients requiring temporal fossa augmentation (population (P)) have high efficacy and safety outcomes?

Exclusion criteria: (1) reviews, in vitro studies, case reports, and literature that is unavailable or incomplete; (2) studies that do not involve human subjects; and (3) articles written in a language other than English.

### 2.3 Data Extraction and Organization

Two blind and independent investigators performed data extraction, and all data were extracted and reviewed by a third investigator based on a pre‐prepared checklist. Disagreements between investigators were discussed and resolved by a third reviewer. study characteristics included the author's name, publication year, study population, mean age, study design, Mean Volume, Filler type, and followed up. The Global Aesthetic Improvement Scale (GAIS) was used to evaluate aesthetic improvement following treatment. GAIS is a validated subjective assessment scale that categorizes cosmetic outcomes from “worse” to “very much improved,” with responders defined as patients rated as “improved” or better. GAIS responder rates were extracted as reported in each study, including physician‐assessed, patient‐assessed, or combined evaluations, depending on the original study design.

### 2.4 Bias Assessment

Two authors independently and blindly assessed the quality of the studies. The Newcastle‐Ottawa Scale (NOS) [16] was used to assess cohort studies and the Cochrane Risk of Bias Tool for Randomized Trials (RoB 2) [17] was used to assess RCTs. The NOS tool evaluates studies based on selection, comparability, and outcome domains, and is scored from 0 to 9, with scores categorized as high (7–9), moderate (4–6), and low risk of bias (0–3). RoB 2 assesses risk of bias across five domains: randomization process, deviations from intended interventions, missing outcome data, outcome measurement, and selection of reported results. Each domain is individually judged as “low risk,” “some concerns,” or “high risk,” and an overall risk of bias judgment is derived from these domain‐level assessments according to the Cochrane guidelines.

### 2.5 Statistical Analysis

Data analyses were conducted using the meta‐analysis packages in STATA/MP.v17 (College Station, Texas, USA). Statistical significance was determined at *p* < 0.05. The I^2^ and τ^2^ statistics were used to assess the degree of heterogeneity between studies. I^2^ values less than 30% indicated minimal or no heterogeneity; I^2^ values between 30% and 60% indicated moderate heterogeneity; and I^2^ values above 60% indicated significant heterogeneity. Publication bias was done via an Egger test and visual inspection of funnel plot asymmetry.

## 3 Results

**FIGURE 1:**
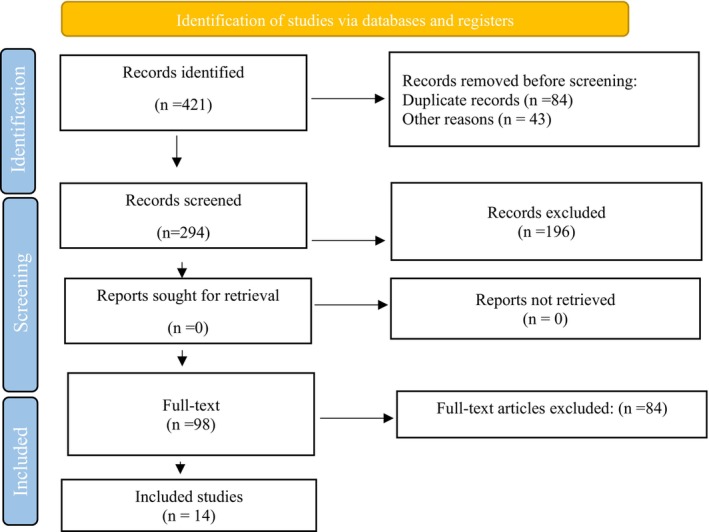
PRISMA 2020 Flow Diagram.

Figure 1 shows the flow chart of article selection. The initial search yielded 421 articles, of which 127 were excluded after title screening based on the study selection criteria. In the next step, after title screening, abstracts of 294 potentially eligible articles were reviewed, and studies that did not meet the inclusion criteria were excluded in this section (*n* = 196). In the next step, by reviewing the full text of 98 articles by two blind and independent authors, the most relevant articles were selected for the purpose of the study, and finally 14 articles were included in the meta‐analysis.

### 3.1 Characteristics of Included Studies

Present meta‐analysis included a total of three RCT studies [18, 19, 20], nine prospective studies [3, 21, 22, 23, 24, 25, 26, 27] and two retrospective studies [28, 29] encompassing 544 patients who received dermal filler to improve temporal fossa. Characteristics of the included studies reported in Table 1.

**TABLE 1:** Baseline characteristics of included studies.

Study. Years	Study design	Number of study population	Mean age	Mean Volume (ml)	Filler type	Followed up (month)
[19]	RCT	205	55	NR	HA	13
[21]	Prospective	12	56.8	1.2	HA	2
[22]	Prospective	25	NR	2	HA	6
[23]	Prospective	22	51.9	2.8	HA	4.5
[18]	RCT	31	54.7	1.1	HA	18
[24]	Prospective	87	49.7	3.5	HA	12
[3]	Prospective	29	52.5	5.3	HA	6
[30]	Prospective	12	36.8	2, 1.5	CaHA, HA	3
[28]	Retrospective	14	50.9	6	HA	1
[20]	RCT	17	46.3	1.42	HA	12
[25]	Prospective	25	NR	1.9	HA	12
[26]	Prospective	20	63.5	3.1	CaHA	12
[27]	Prospective	15	37.1	1.3	HA	1
[29]	Retrospective	20	46	1.9	HA	9

Abbreviations: CaHA: calcium hydroxyapatite; HA: hyaluronic acid.

### 3.2 Risk of Bias

The randomization process was judged to be at low risk of bias in all studies. However, deviations from intended interventions were rated as “some concerns” in all trials, mainly due to the lack of blinding inherent to aesthetic injection procedures. Missing outcome data, outcome measurement, and selective reporting domains were generally assessed as low risk across all included studies. Overall, all RCT studies were judged to have a low risk of bias according to the Cochrane RoB 2 tool (Table 2). Overall, all included cohort studies demonstrated high methodological quality, with total scores ranging from 7 to 9 (Table 3).

**TABLE 2:** Cochrane Collaboration's tool for assessing the risk of bias of RCTs.

Study	Randomization process	Deviations from intended interventions	Missing outcome data	Outcome measurement	Selection of reported result	Overall risk of bias
[19]	Low	Some concerns	Low	Low	Low	Low
[18]	Low	Some concerns	Low	Low	Low	Low
[20]	Low	Some concerns	Low	Low	Low	Low

**TABLE 3:** Newcastle–Ottawa scale for assessing the risk of bias of cohort studies.

Study	Selection (max 4★)	Comparability (max 2★)	Outcome (max 3★)	Total
[21]	★★★★	★	★★★	8
[22, 31]	★★★★	★	★★★	8
[23]	★★★★	★	★★★	8
[24]	★★★★	★	★★	7
[3]	★★★★	★	★★★	8
[30]	★★★★	★	★★★	8
[28]	★★★★	★	★★★	8
[25]	★★★★	★	★★	7
[26]	★★★★	★	★★	7
[27]	★★★★	★	★★★	8
[29]	★★★★	★	★★	7

*Note:* Each star represents one point awarded.

### 3.3 Effectiveness

#### 3.3.1 GAIS Responder Rate

**FIGURE 2:**
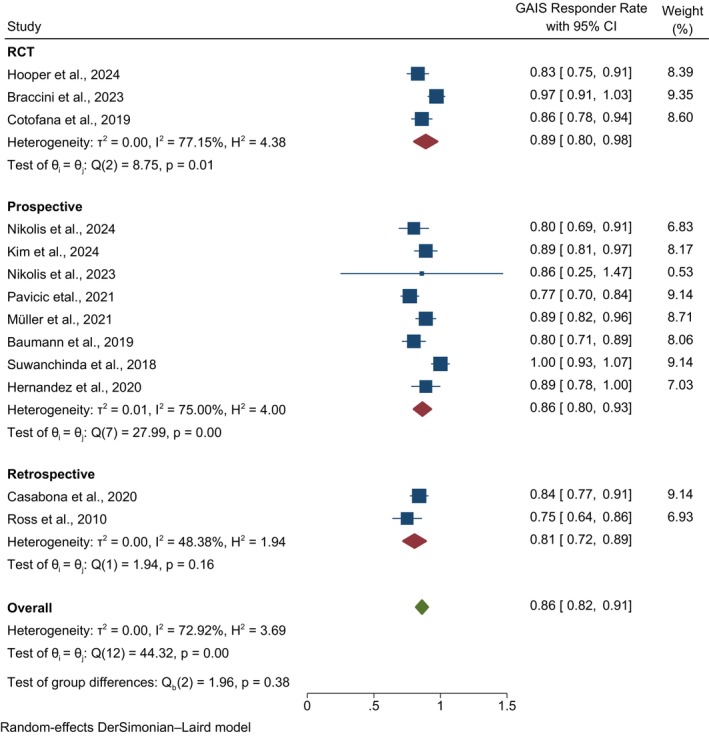
Forest plot showing pooled GAIS responder rates for hyaluronic acid (HA) fillers stratified by study design (RCTs, prospective, and retrospective studies) using a DerSimonian–Laird random‐effects model.

Subgroup meta‐analysis stratified by study design showed consistent results across study types. For HA fillers, the pooled GAIS responder rate was 86.5% (ES: 0.865; 95% CI: 0.799–0.930) in prospective studies, 89.0% (ES: 0.890; 95% CI: 0.801–0.978) in RCTs, and 80.5% (ES: 0.805; 95% CI: 0.720–0.891) in retrospective studies (Figure 2). Although heterogeneity was observed within study subgroups, the between‐group difference was not statistically significant (Qb = 1.96, *p* = 0.376), suggesting that study design did not significantly influence the overall effect size. The overall pooled GAIS responder rate for HA fillers remained 86.1% (ES: 0.861; 95% CI: 0.816–0.906), with substantial heterogeneity (I^2^ = 72.92%).

For CaHA fillers, two prospective studies were included in the meta‐analysis. The GAIS responder rate was 84.1% (ES: 0.841; 95% CI: 0.723–0.958). High heterogeneity was observed across studies (I^2^ = 73.10%, τ^2^ = 0.0053, *p* = 0.054).

**FIGURE 3:**
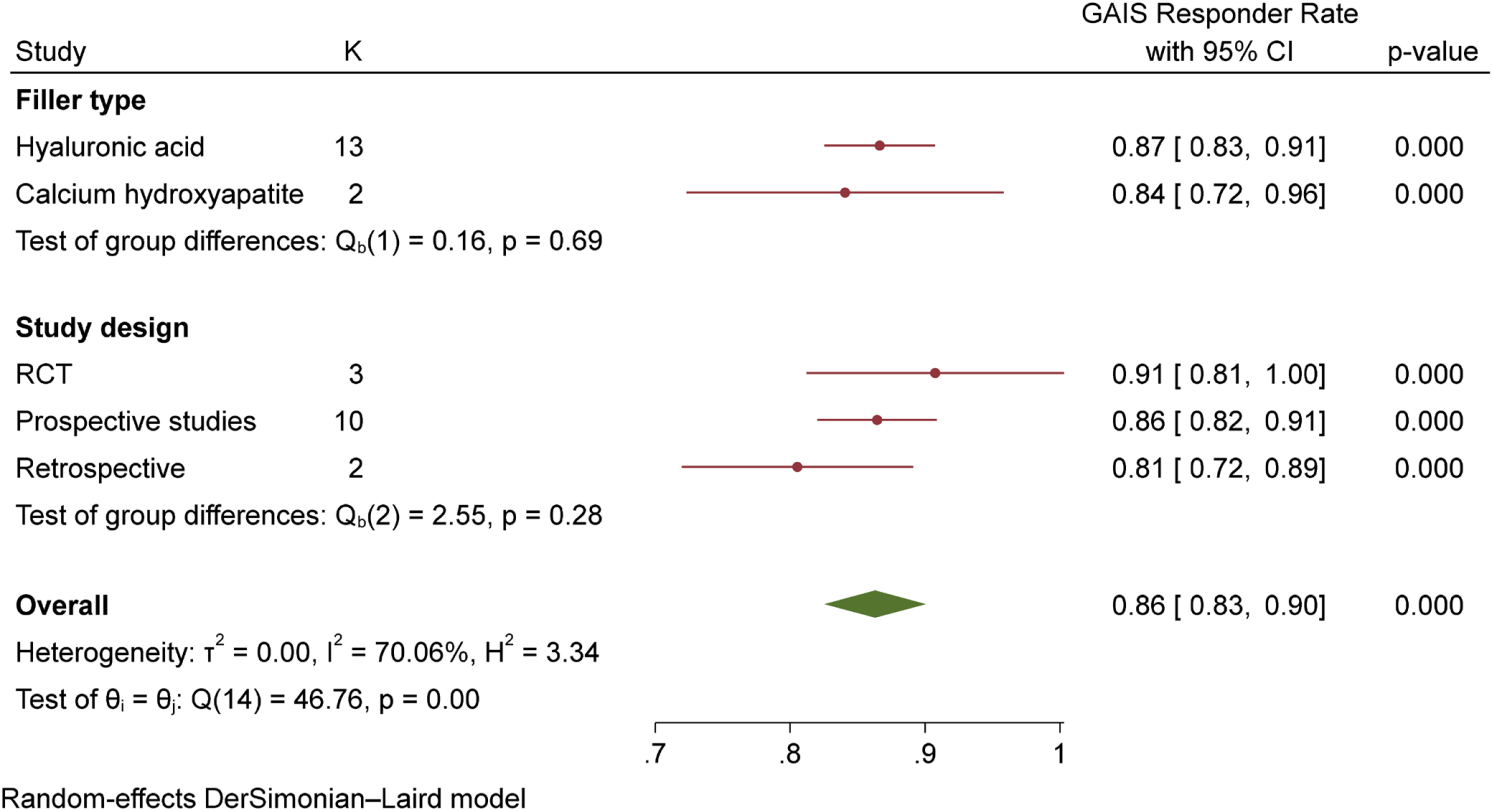
Forest plot showing pooled GAIS responder rates stratified by filler type (HA vs. CaHA) and study design, using a random‐effects model.

Subgroup meta‐analysis based on filler type demonstrated no statistically significant difference between HA and CaHA fillers (Qb = 0.16, *p* = 0.687) (Figure 3), indicating comparable GAIS responder rates. Similarly, subgroup analysis based on study design showed no significant differences among randomized controlled trials, prospective studies, and retrospective studies (Qb = 2.55, *p* = 0.280), suggesting that study design did not significantly influence the pooled outcomes. The overall pooled GAIS responder rate was 86.3% (95% CI: 82.5–90.1), with substantial heterogeneity (I^2^ = 70.06%) (Figure 3).

#### 3.3.2 Meta Regression

According to meta‐regression, the effectiveness of dermal fillers was not related to age, and acceptable effectiveness was observed in older ages. Also, the follow‐up period did not significantly affect the effectiveness results, while the injection rate showed a significant relationship between the effectiveness results in temporal fossa improvement (Table 4).

**TABLE 4:** Meta regression.

_meta_es	Coefficient	Std. err.	z	*p*‐value	[95% conf. interval]	
					Lower	Upper
age	0.0019	0.002	0.75	0.451	−0.0031	0.0071
Volume	−0.004	0.001	−2.35	0.019	−0.0084	−0.0007
Follow‐up	−0.0065	0.007	−0.90	0.366	−0.0208	0.0076
_cons	1.0986	0.0808	13.59	0.000	0.94019	1.2570

#### 3.3.3 Safety/Adverse Events

Reported adverse events across the included studies were generally mild and transient. The most commonly reported complications included edema, bruising, tenderness, erythema, and temporary swelling at the injection site. No study consistently reported severe or irreversible adverse events such as skin necrosis or vision loss following temporal filler injection.

#### 3.3.4 Publication Bias

**FIGURE 4:**
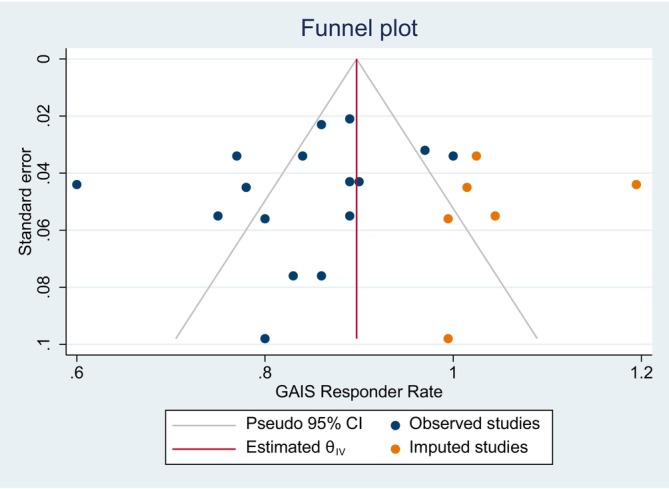
Funnel plot of publication bias.

In the funnel plot, a symmetrical distribution of studies was observed on both sides of the mean effect, indicating the absence of publication bias. Also, the results of the Egger test (*p* > 0.05) test statistically confirmed the absence of publication bias (Figure 4).

## 4 Discussion

A thorough understanding of the basic anatomy, age‐related changes, and the complex process of injecting dermal fillers into the temporal region is essential. The goal of these injections, in addition to filling in sunken areas, is to create a lifting effect that revitalizes the face and restores its youthful appearance [11]. The present study attempted to evaluate the effectiveness of various dermal fillers for temporal fossa augmentation; most studies evaluated the effectiveness of HA fillers; only two studies were found that evaluated the effectiveness of CaHA and one study of allograft adipose matrix. No studies were found that evaluated PLLA or PMMA. The results of the present study showed that the effectiveness of HA fillers for temporal fossa augmentation is higher than that of other dermal fillers. However, the heterogeneity between studies was very high, so the results of the present study should be interpreted with caution; the reason for this heterogeneity could be related to the methodology of the studies; Meta‐regression also showed that there is a significant relationship between the volume of injected fillers and effectiveness, and the difference in the volume of injected fillers can be considered one of the important reasons for the heterogeneity between studies.

The concentration of neurovascular structures in the temporal region also poses risks. Vital structures and the temporal branches of the facial nerve are found in the anterior half of the inferior temporal chamber, known as the “zone of caution.” Clinicians should exercise caution when injecting into this area because damage to these structures can cause serious complications, such as vascular and nerve damage([32]; [31]).

The temporal region consists of multiple anatomical layers [33], and injection planes were not consistently reported across included studies. Most papers describe deep injections between the temporalis muscle and the temporal bone. However, recent anatomical evidence suggests that this space may not represent a true potential plane, and in many cases the filler is likely deposited within the temporalis muscle [34]. This is clinically important, as muscle contraction during mastication may theoretically increase the risk of filler compression or migration. For this reason, deep injections in this plane are now considered less favorable, and more anatomy‐guided approaches are recommended.

Another important consideration in temporal filler augmentation is the rheological properties of the injected material [35]. The temporal region is a multilayered anatomical area exposed to dynamic muscular forces, particularly from the temporalis muscle. Therefore, fillers with higher elasticity and structural stability may theoretically provide better resistance to tissue compression and maintain longer‐lasting volume restoration. However, rheological characteristics were not consistently reported across the included studies, and therefore could not be analyzed in a quantitative manner. This represents an important factor that should be considered in future clinical studies and treatment selection.

Another important factor in temporal filler injection is the selection of the cannula entry site. The medial aspect of the hairline and the intersection of the temporal line is the recommended entry point for safe and effective injection. This location reduces the risk of damage to critical structures and allows for precise placement of the filler in both the deep and superficial areas. Clinicians can reduce the risk of complications by selecting the appropriate entry point to ensure accurate and safe filler injection [36, 37]. A visual confirmation of the lifting effect that can be achieved with filler injection is the upward displacement of the superficial layer of the DTF, often shown on imaging studies as a “green line.” Clinical practice should focus on this anatomical feature because it is essential for the success of temporal filler injection [38].

According to the results of the present study, the high effectiveness of dermal fillers in Temporal Fossa Augmentation was evident; however, the present study had some limitations and high heterogeneity was observed between studies; firstly, the sample size of the studies was very small and there are not many studies on the use of dermal fillers other than HA for Temporal Fossa Augmentation, and this cannot be considered a good comparison; therefore, larger sample sizes and further research are needed to confirm the safety and effectiveness of injectable fillers. Some studies used ultrasound imaging and photographic comparisons to demonstrate the lifting effect, and some evaluated objective quantitative measurements such as biomechanical assessments or 3D volumetric analysis, which could be a reason for the heterogeneity. Also, a limited number of studies have examined the long‐term effects of dermal fillers on the temporal fossa, and long‐term follow‐up periods should be conducted to assess the durability of the lifting effect and its possible complications. Injection technique may substantially influence both aesthetic outcomes and complication rates in temporal filler augmentation. Variations in injection plane, cannula versus needle use, and filler placement strategy were inconsistently reported across studies, preventing subgroup analysis. Additionally, ethnicity and gender‐related anatomical differences were not adequately reported in the included studies, limiting the generalizability of the findings. The substantial variation in injected filler volume across studies may be attributed to differences in the severity of temporal hollowing, filler characteristics, injection techniques, aesthetic goals, and physician preference. In addition, individual anatomical variation and unilateral versus bilateral treatment approaches may have contributed to the heterogeneity in injection volume. In the present study, meta‐regression demonstrated a significant association between injection volume and treatment effectiveness. A limitation of this study is the heterogeneity in GAIS assessment methods across included studies, including variability in responder definitions and evaluator type (physician, patient, or combined), which may have affected the comparability of pooled results.

## 5 Conclusion

Temple volume augmentation using injectable soft tissue fillers appears to be an effective minimally invasive procedure for restoring temporal hollowing and improving aesthetic outcomes. However, due to limited and heterogeneous reporting of safety outcomes across the included studies, conclusions regarding overall safety should be interpreted with caution. The temporal region is anatomically complex, and successful outcomes depend on appropriate injection techniques and a thorough understanding of facial anatomy to minimize potential complications. Further well‐designed randomized controlled trials with standardized reporting of both efficacy and safety outcomes are required to optimize treatment protocols and injection strategies.

## Conflicts of Interest

The authors declare no conflicts of interest.

## Data Availability

The data that support the findings of this study are available from the corresponding author upon reasonable request.
